# Lack of association of the *WRN* C1367T polymorphism with senile cataract in the Israeli population

**Published:** 2010-08-28

**Authors:** M. Ehrenberg, O. Dratviman-Storobinsky, B.R. Avraham-Lubin, N. Goldenberg-Cohen

**Affiliations:** 1 Department of Ophthalmology, Rabin Medical Center, Petach Tikva, Israel; 2 Sackler School of Medicine, Tel Aviv University, Tel Aviv, Israel; 3 The Krieger Eye Research Laboratory, Felsentein Medical Research Center, Petach Tikva, Israel; 4 Department of Ophthalmology, Pediatric Division, Schneider Children’s Medical Center of Israel, Petach Tikva, Israel

## Abstract

**Purpose:**

Werner syndrome is an autosomal recessive disease of premature aging caused by a polymorphic C1367T mutation in the Werner (*WRN*) gene. Although there are differences between the pathobiology of normal aging and the phenotype of Werner syndrome, the clinical age-related changes are similar. The aim of the study was to investigate the incidence of the C1367T (rs1346044) polymorphism in patients with age-related cataract.

**Methods:**

The study group consisted of 81 patients with senile cataract undergoing cataract extraction surgery. Data on age, sex, and medical history of microvascular disease and cancer were obtained from the medical files. Anterior lens capsule material was collected during surgery. DNA was extracted, amplified by polymerase chain reaction, and screened for the C1367T polymorphism in *WRN* using restriction enzymes followed by sequencing.

**Results:**

There were 33 male and 48 female patients of mean age 74.3±9 years. Genotypic frequencies were 67% for TT and 33% for TC. None of the patients had the CC genotype. Ten patients had a history of myocardial infarct, 8 cerebrovascular accident, and 8 various tumors. The distribution of these morbidities was similar in the two genotype groups.

**Conclusions:**

The distribution of the C1367T *WRN* polymorphism in patients with senile cataract is similar to that in the normal population. Cataract formation in the elderly is not linked to a *WRN* mutation.

## Introduction

Progeria is a rare group of diseases with striking features that resemble accelerated aging [1].

Werner syndrome is a less well known but more common form of progeria, with a frequency of 1×10^5^-1×10^7^ depending on the geographic area [2]. Also termed progeria of adults, Werner syndrome first becomes apparent in puberty (mean age) with growth arrest and thinning and graying of the hair. Other manifestations include skin wrinkling, cataract, osteoporosis, and premature arteriosclerotic disease that leads to heart attacks and strokes [3-5]. Werner syndrome has also been associated with various types of cancer [6]. Many laboratory abnormalities have been reported [7]. The syndrome is caused by a polymorphic C1367T mutation in the Werner gene (*WRN)* located on the short arm of chromosome 8.

*WRN* encodes a multifunctional nuclear protein of the RecQ family which functions as an exonuclease and endohelicase [8-12] with apparent involvement in transcriptional and chromosomal segregation and DNA repair/recombination [13]. Mutations in *WRN* lead to a loss of function of the protein and a breakdown in genome integrity [14]. Although the genetic basis of Werner syndrome is unknown, the inheritance pattern and paternal age effect, in addition to the absence of findings of consanguinity, point to a sporadic dominant mutation.

Diseases of premature aging have prompted interest among geneticists because the study of their underlying mechanisms can provide insights not only into these rare disorders themselves but also into the normal aging process [1,15]. While the phenotype of Werner syndrome differs from the pathobiology of normal aging, the clinical age-related changes are similar. To date, researchers have investigated the possible relationship of many mutations, deletions, and polymorphisms of the *WRN* gene to such age-related diseases as cardiovascular disease [6], hypertension, diabetes mellitus, dementia, osteoporosis [3], and some cancers [16-19]. In the eye, the literature has focused on complications after cataract surgery [20,21]; one case of a dislocated lens to the vitreous has been reported as well [22]. Although bilateral cataract develops early in patients with Werner syndrome [7], and other laminopathies have been linked with congenital cataract [23-26], to our knowledge there are no studies of possible protein changes in the lens as a result of progeroid mutations or a link between senile cataract and abnormalities in *WRN*.

The aim of the present study was to investigate a possible role of the C1367T (rs1346044) polymorphism in the *WRN* gene in age-related cataract in an Israeli population.

## Methods

### Patients

The study group included 81 patients undergoing routine cataract surgery at a major tertiary center. The study was approved by the institutional and national review boards, and all patients signed an informed consent form.

All patients were examined preoperatively by slit-lamp, and cataract type and grade were categorized. Background data were derived from the medical files. Anterior lens capsule material excised during surgery (one sample per patient) was analyzed for the C1367T polymorphism in the *WRN* gene.

### DNA Isolation

The capsules containing single-layer lens epithelial cells were suspended in 5 ml of conservation medium until isolation of genomic DNA. DNA was extracted using standard sodium dodecylsulfate (SDS)/proteinase K digestion followed by phenol-chloroform extraction and ethanol precipitation.

### C1367T polymorphism in the *WRN* gene

A DNA sequence of 195 bp, which contains the polymorphic site, was amplified by polymerase chain reaction (PCR) using the following primers: 5′-GCC TAA TCA GAA TGT TAG TT-3′ and 5′-CCT CAG TAT TGA TGC CTA CTT C-3′. Amplification was performed in a 50-µl reaction volume containing 100 ng of sample DNA as a template. The PCR parameters were as follows: denaturation at 95 °C for 3 min, 35 cycles of 45 s at 95 °C, annealing at 58 °C for 45 s, and extension of 1 min at 72 °C with Taq polymerase. The PCR product was amplified on 2% agarose gel and visualized with ethidium bromide staining.

**Figure 1:**
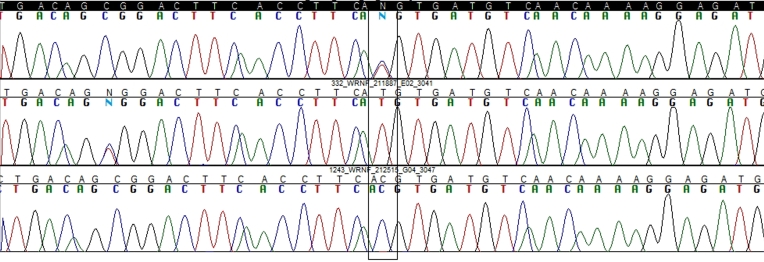
DNA sequencing. CT, TT, and CC polymorphism is demonstrated, respectively. The later is for demonstration, not from the study population.

Direct sequencing of the PCR products was performed for selected samples, with Big Dye Terminator Cycle Sequencing reagents using the ABI PRISM 3700 DNA Analyzer (Applied Biosystems, Foster City, CA; Figure 1).

**Figure 2:**
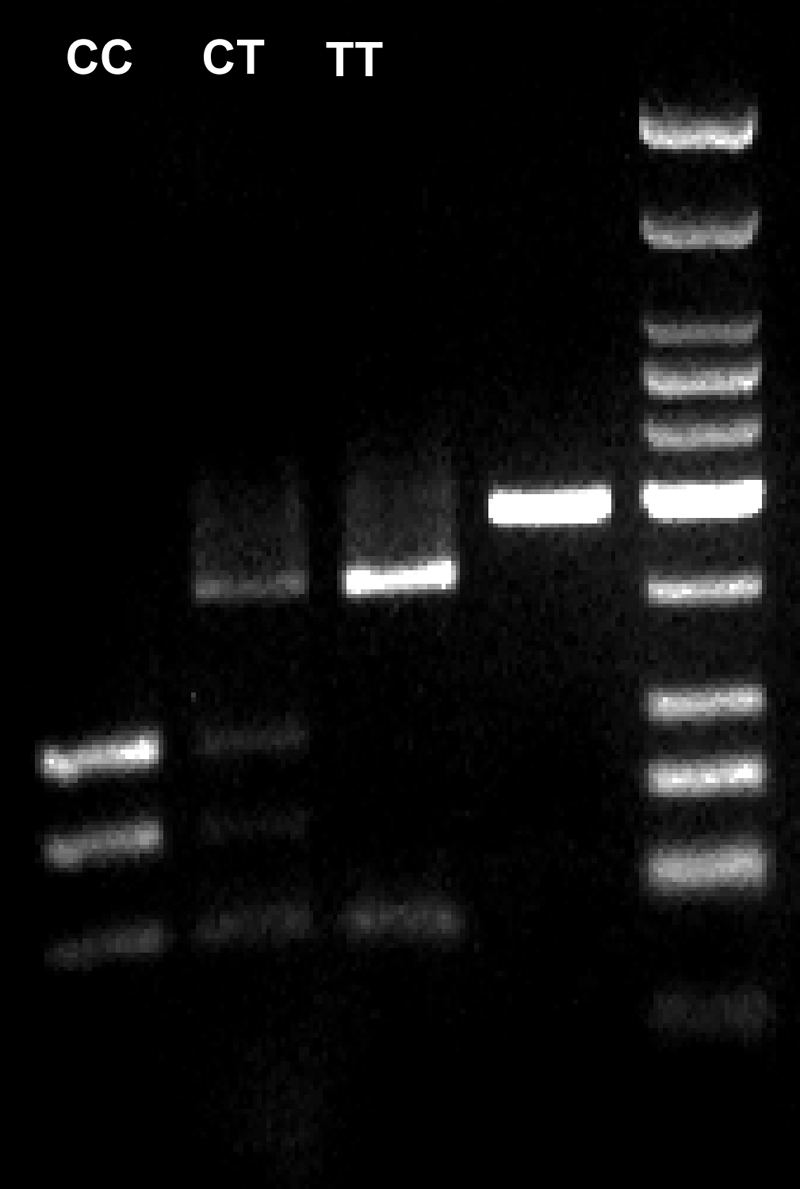
The C1367T polymorphism. Following the use of restriction enzymes, the gel shows: 1, DNA ladder; 2, PCR product well, demonstrating a 195 bp length sequence; 3, TT well, demonstrating two lengths of DNA of 158 bp and 37 bp; 4, CT well, demonstrating 4 DNA lengths of 158 bp, 93 bp, 65 bp, and 37 bp; 5, CC well, demonstrating 3 DNA lengths of 93 bp, 65 bp, and 37 bp (example taken as control).

The T→C alteration in *WRN* results in the loss of a BsaA1 restriction site in the abnormal sequence. Therefore, upon digestion with BsaA1 (R0531; New England Biolabs, Beverly, MA), the normal sequence yielded two fragments of 158 bp and 37 bp, whereas the C-altered sequence yielded three fragments of 93 bp, 65 bp, and 37 bp, which were separated on a 4% agarose gel (Figure 2).

### Statistical analysis

The results were statistically analyzed with SPSS for Windows, version 15.0.1 (SPSS- Inc, Chicago, IL): A p value of less than 0.05 was considered statistically significant.

Between-group differences in cataract type and grade were analyzed by the χ^2^ test; in sex and underlying diseases, by Fisher exact test; in age, by *t*-test. Correlations between variables were analyzed by Pearson correlation test.

## Results

Eighty-one patients participated in the study, 33 male (41%) and 48 female (59%), of mean (±SD) age 74.3±9.6 years (range: 52–93 years). Other morbidities included hypertension 66.7%, diabetes mellitus 36.1%, dyslipidemia 48.6%, s/p myocardial infarction 14.1%, s/p cerebrovascular accident 11.3%, and cancer 11.3% (colorectal carcinoma, 2.4%, including one patient also with prostate carcinoma; breast cancer, 2.4%, including one patient also with uterine carcinoma; transitional cell carcinoma of the bladder, 1.2%; synovial sarcoma, 1.2%).

Analysis of allele frequencies of the *WRN* polymorphism revealed the CT genotype in 33.3% of patients, and the TT genotype, in 66.67%. No homozygosity for CC was found. There was no association of genotype polymorphism with the presence or stage of cataract. The distribution of cataract and other morbidities by genotype is shown in Table 1.

**Table 1:** WRN allele polymorphism, cataract grading and systemic co-morbidities.

** **	**Gene polymorphism**		** **
**Disorder**	**C/T n=27 (33%)**	**T/T n=54 (66.7%)**	**Statistical significance**
Nuclear cataract			
Stage 0	1	4	NS
Stage 1	9	28	NS
Stage 2	14	24	NS
Stage 3	8	9	NS
Stage 4	3	3	NS
Hypertension	70	65	NS
Diabetes mellitus type 1	39	35	NS
Dyslipidemia	38	54	NS
Myocardial infarct	16	13	NS
Cerebro-vascular accident	4	7	NS
Tumor	12	11	NS

In the Table, “NS” indicates not statistically significant.

## Discussion

The present study investigated the C1367T *WRN* polymorphism and genotype frequencies in elderly patients with senile cataract. We found that the genotype distribution was similar to that reported in the general population [26-29], namely 2/3 TT and 1/3 CT. No association was found between the genotype and type or stage of cataract or other age-related morbidities.

Molecular studies strongly suggest that aging is triggered by two mechanisms: DNA damage and telomere shortening [30]. In the first, the cumulative DNA damage accompanied by DNA repair deficiencies results in genomic instability and accelerated cellular senescence. Aging due to both mechanisms is strongly dependent on the TP53 (p53) status.

The *WRN* gene is considered the “caretaker” of the genome [31], with the protein serving as an important link between repair of defective DNA and processes related to aging. The gene is expressed within the central nervous system and throughout the brain, and is present in both neurons and glia. Analysis of *WRN* RNA levels throughout the life cycle revealed the highest levels in embryonic brain tissue and a biphasic pattern of expression from the early postnatal period into adulthood. Mutations in *WRN* are believed to result in the deleterious loss of normal WRN function.

The eye is part of the central nervous system. The transparency of the lens is maintained by a specific mechanism of nuclear differentiation and elimination of the lens fiber cells followed by apoptosis [10]. The purpose of the lens denucleation is to reduce light scatter. Studies of normal aging of the lens suggest that transcriptional shutdown precedes laminar reorganization and chromatin breakdown during lens fiber cell denucleation [15].

Senile cataract, which disrupts normal lens denucleation, is one of the most common age-related disorders. It manifests early in most patients with Werner syndrome, a classic progeroid premature-aging syndrome caused by a single-gene mutation. Although the function of *WRN* has been intensively investigated in primary fibroblast and fibroblast cell lines, little is known about the normal expression pattern of the protein in the eye. We speculated that a search for the C1367T *WRN* polymorphism responsible for Werner syndrome in elderly patients with cataract might shed light on the molecular basis of both the disease and the normal aging process of the eye [32]. However, we did not identify any homozygosity of the CC alleles, and the distribution of the heterozygous polymorphism was similar to that found in the general Israeli population (~33%). Cataract, and other age-related diseases, were not linked to a mutation of the *WRN* gene.

These results are in line with the larger Elderly Brazilian Longitudinal Study [26], wherein no association was found between *WRN* C1367T and cardiovascular diseases, diabetes mellitus type 2, obesity, dementia, depression, and neoplasms. In addition, Bohr et al. [27], in the Baltimore Longitudinal Study of Aging, failed to show any influence of the *WRN* polymorphism on coronary artery disease. However, studies in Japanese populations found that patients homozygous for TT were at nearly threefold higher risk of myocardial infarct than the general population [33]; other researchers from countries other than Japan reported a similar risk for CT in their populations [34]. CC homozygosity posed a lower risk [34,35] and also protected against the development of type 2 diabetes mellitus [36]. In the present study, in which none of the patients had the CC genotype, the distribution of type 2 diabetes was similar in patients with TT and TC.

Studies of central nervous system diseases in this context yielded no association of the *WRN* polymorphism with increased risk of either Alzheimer disease [28] or gliomas [29]. Breast cancer was not associated with C1367T, but it was associated with another polymorphism of the *WRN* gene, A46729C [37].

The lack of an association of the *WRN* C1367T polymorphism and senile cataract in the present study could suggest that abnormalities in *WRN* in position C1367T do not lead to abnormal lens fiber cell denucleation in adults or that the influence of the protein on lens laminopathies is not crucial to the lens aging process. The lack of association with other age-related morbidities might distinguish Israelis from Japanese and other populations, or it might be explained by the limited number of participants in our sample. Additional studies are needed to further investigate possible links of *WRN* polymorphisms with age-related diseases, including cataract.
